# Evaluating remote sensing datasets and machine learning algorithms for mapping plantations and successional forests in Phnom Kulen National Park of Cambodia

**DOI:** 10.7717/peerj.7841

**Published:** 2019-10-22

**Authors:** Minerva Singh, Damian Evans, Jean-Baptiste Chevance, Boun Suy Tan, Nicholas Wiggins, Leaksmy Kong, Sakada Sakhoeun

**Affiliations:** 1 Imperial College, Centre of Environmental Policy, London, United Kingdom; 2 École Française d’Extrême-Orient, Paris, France; 3 Phnom Kulen Program, Archaeology and Development Foundation, London, United Kingdom; 4 Angkor International Research and Documentation Centre, Siem Reap, Cambodia, Siem Reap, Cambodia; 5 School of Earth and Environmental Sciences, University of Queensland, St Lucia, Australia

**Keywords:** Tropical forests, Deforestation, Plantations, SE Asia, Remote sensing, Landsat, ALOS PALSAR, Machine learning, LiDAR, Support vector machines

## Abstract

This study develops a modelling framework by utilizing multi-sensor imagery for classifying different forest and land use types in the Phnom Kulen National Park (PKNP) in Cambodia. Three remote sensing datasets (Landsat optical data, ALOS L-band data and LiDAR derived Canopy Height Model (CHM)) were used in conjunction with three different machine learning (ML) regression techniques (Support Vector Machines (SVM), Random Forests (RF) and Artificial Neural Networks (ANN)). These ML methods were implemented on (a) Landsat spectral data, (b) Landsat spectral band & ALOS backscatter data, and (c) Landsat spectral band, ALOS backscatter data, & LiDAR CHM data. The Landsat-ALOS combination produced more accurate classification results (95% overall accuracy with SVM) compared to Landsat-only bands for all ML models. Inclusion of LiDAR CHM (which is a proxy for vertical canopy heights) improved the overall accuracy to 98%. The research establishes that majority of PKNP is dominated by cashew plantations and the nearly intact forests are concentrated in the more inaccessible parts of the park. The findings demonstrate how different RS datasets can be used in conjunction with different ML models to map forests that had undergone varying levels of degradation and plantations.

## Introduction

Conversion of forests to plantations for commodity production is a leading cause of permanent forest loss globally. Analysis of satellite imagery from 2000–2015 revealed that approximately 27% of global forest loss could be attributed to plantation creation (Curtis et al., 2018). The forests of tropical Asia, and especially lowland forests of insular Southeast Asia, are the worst affected by the global demand for commodities (such as oil palm and rubber) and the subsequent conversion of forests to agricultural plantations (Koh & Wilcove, 2008; Edwards et al., 2010; Edwards et al., 2013). Commercial agricultural plantations such as rubber and acacia are also encroaching in the forests of continental Asia (Torbick et al., 2017; Zeng et al., 2018). Plantations for commodity crops now exist in Laos, China, Vietnam and Myanmar (Phompila et al., 2014; Grogan et al., 2015; Chen et al., 2016).

In addition to plantations, many tropical Asian ecosystems are significantly dominated by successional forests (Singh et al., 2014); forests that regrow after forest clearance or deforestation (Lu et al., 2014). While the role of primary tropical forests in preserving biodiversity is well-established, logged and degraded forests too can provide a valuable reservoir for endangered biodiversity and carbon stocks (Woodcock et al., 2011; Edwards & Laurance, 2013; Mukul, Herbohn & Firn, 2016; Lennox et al., 2018). Thus, the retention of regenerating and logged forests has been recommended as a means of preventing a biodiversity collapse in SE Asia (Wilcove et al., 2013). However, the ability of successional forests to provide habitat for species and store carbon depends on the age, structure and level of degradation of the forest (Edwards et al., 2011; Singh et al., 2014). Mapping the spatial extent of plantations and differentiating between forests of different successional ages and ecological conditions is important for the conservation of Asia’s tropical forests.

Remote sensing (RS) data have been extensively used for mapping tropical ecosystems. Landsat data which can go back to the mid-1970s with spatial resolution of 30-m have been used extensively to map land use types and changes in the tropics (Grogan et al., 2015; Maryantica & Lin, 2017; Midekisa et al., 2017). Landsat data have proven useful in mapping and monitoring forest disturbance and recovery at global and continental scales (Gong et al., 2013; Midekisa et al., 2017; DeVries et al., 2015; Liu et al., 2017a). Single-date and multi-temporal Landsat data have helped map accurately the extent of plantations and successional forests in the tropics (Connette et al., 2016; Kelley, Pitcher & Bacon, 2018). Conventional approaches for using remote sensing data require users to download and process several terabytes of data. This is time-consuming and often out-of-the-reach for many practitioners. Google Earth Engine (GEE) is a cloud-based platform which allows the user to process RS data, including Landsat data using inbuilt pre-processing algorithms (Gorelick et al., 2017). Landsat data obtained via the GEE platform have been used to quantify land cover types and changes at different scales and in different ecosystems (Midekisa et al., 2017; Tsai et al., 2018).

Over the past few years, Landsat data were also used in conjunction with machine learning (ML) algorithms to distinguish between and map different land use types, including natural forests, anthropogenic land use types (including plantations) and degraded forests (Sesnie et al., 2010; Connette et al., 2016). ML algorithms were also implemented on GEE obtained Landsat data to map forest types, examine spectral properties and delineate shade-grown coffee in Nicaragua (Kelley, Pitcher & Bacon, 2018) and the oil palm plantations in Indonesia (Lee et al., 2016). Some of the most common machine learning techniques for carrying out land cover classification and mapping include, support vector machines (SVM), random forests (RF) and artificial neural networks (ANN). These have been implemented in a variety of different settings, including mapping heterogenous tropical landscapes (Sesnie et al., 2010; Szuster, Chen & Borger, 2011; Gong et al., 2013; Paneque-Gálvez et al., 2013; Shiraishi et al., 2014; Chuang & Shiu, 2016; Thanh Noi & Kappas, 2018; Hartling et al., 2019). The ability of these common ML algorithms to distinguish between and map forests that have undergone varying levels of degradation and plantations in the human-modified forests of the Greater Mekong region countries needs to be evaluated further for the different forest ecosystems of the region in order to support conservation management.

The humid tropics suffer from the problem of persistent cloud cover which impedes the efficacy of optical RS data sources such as Landsat (Asner, 2001). This makes synthetic aperture radar (SAR) data increasingly being used in conjunction with optical data for land cover mapping in the tropics since it is relatively unaffected by cloud cover. Moreover, SAR data offer an additional benefit of having the ability to retrieve forest structure information (Mitchard et al., 2012). While it is possible to use Landsat and SAR data separately for land cover mapping, researches have shown that combining these datasets produce more accurate land cover maps in tropical ecosystems (Lu et al., 2011; Lu et al., 2014). LiDAR derived data also provides valuable information about the forest vertical structure that are useful in distinguishing different successional stages of regenerating tropical forests (Marselis et al., 2018). LiDAR data have been used with both Landsat and ALOS data to identify varying levels of forest degradation in Laos (Singh et al., 2017a) and for distinguishing between natural forests and cashew plantations in Cambodia (Singh et al., 2018).

In this study, we evaluate the ability of some common RS data and ML algorithms tools in mapping human modified forest-plantation tropical ecosystems, focusing on the cashew plantations that have encroached in an IUCN Category II protected forests of Phnom Kulen National Park (PKNP) in Cambodia. Despite its protected status, PKNP has experienced high deforestation and degradation rates. As with other protected areas in Cambodia, faces significant threats from local resource extraction activities (Singh et al., 2018). This research seeks to: (a) examine the efficacy of different RS data sources: Landsat, ALOS and LiDAR (either alone or in conjunction with each other) to delineate the different land cover types; (b) compare the performance of three commonly used for land cover classifications ML algorithms (SVM, RF and ANN) in delineating and mapping forests that have undergone varying levels of degradation and cashew plantations; and (c) assess the structural and spectral properties of the different land use types in the area.

## Materials & Methods

### Study Area

**Figure 1:**
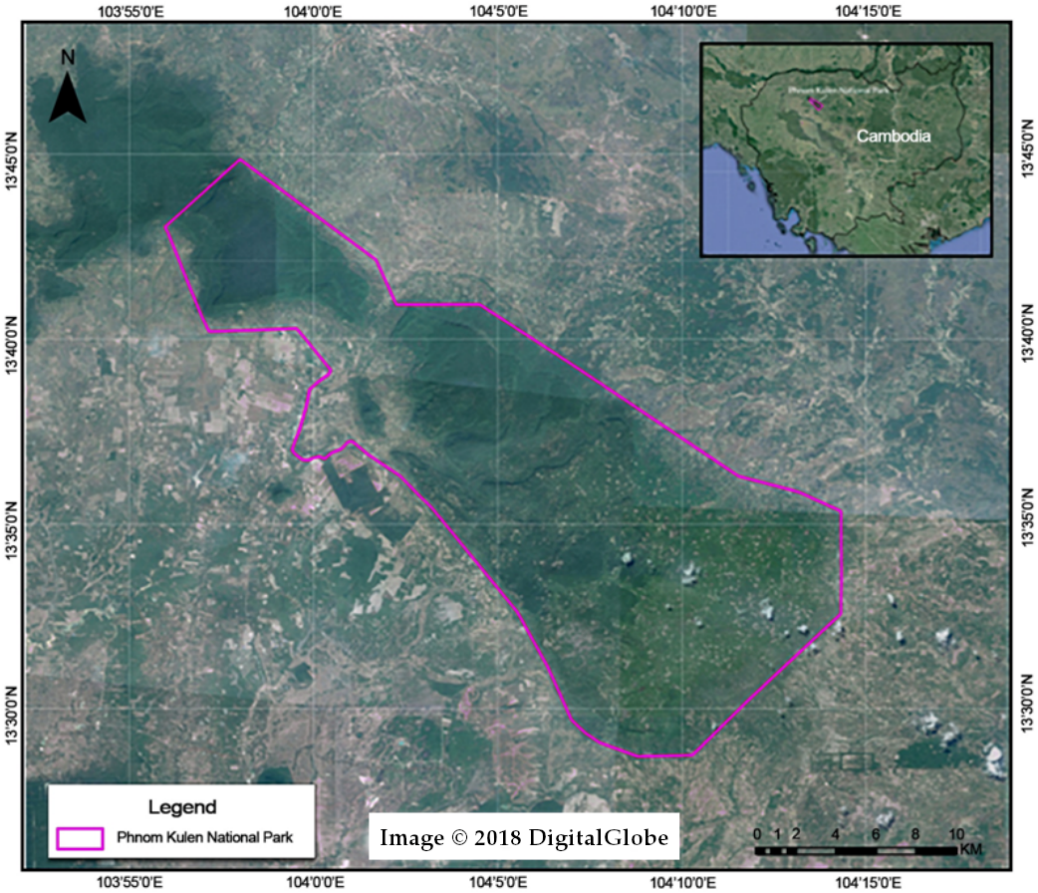
Location of the Phnom Kulen National Park (PKNP) in Cambodia.

PKNP, mainly dominated by semi-evergreen forests, is located in Siem Reap Province in north-western Cambodia (Fig. 1). At 37,380 hectares, it covers the entire Kulen plateau which is a critical area for biodiversity and a significant component of the regional watershed. It is comprised mostly of regenerating and secondary forests along with severely degraded forests, cassava and cashew plantations, mostly located in the southern part (Singh et al., 2018).

Table 1 shows seven main land use classes identified in the area (Singh et al., 2017a; Sirro et al., 2018). The data pertaining to the geo-location of these land use types were collected in the field using a handheld GPS. These geo-locations were then verified using high-resolution imagery available on Google Earth.

**Table 1:** Land use/cover types and their characteristics.

**Land use/cover type**	**General characteristics**
Cashew Plantations	Cashew monocultures (created as result of clear felling forests).
Lightly Logged Forests	There was no previous record of these forests having undergone extensive selective logging and/or resource extraction. Qualitative field monitoring by JBC indicates light selective logging may have started occurring after 2015.
Degraded Forests	These forests have undergone several rounds of logging and resource extraction. These are mostly scrub forests with less than 50% canopy cover.
Selectively Logged Forests	These forests had a greater than 50% forest cover but most of the large Dipterocarp and rosewood trees had been selectively logged. Selective logging of these species was ascertained by the presence of tree stumps and confirmed by the forest rangers.
Regenerating for More Than 10 Years	These forests were clear felled/burnt for agricultural use more than a decade ago and were regenerating for more than 10 years as of March 2016.
Regenerating for Less Than 10 Years	These forests were clear felled/burnt for agricultural use less than a decade ago and were regenerating for less than 10 years as of March 2016.
Bare Earth/New Cassava Plantations	Cassava plantations (as of 2016) which had the crops, scrubby vegetation and bare earth patches.

### Remote sensing data

#### Landsat 8 data

The Landsat 8 Operational Land Imager TOA (top-of the-atmosphere) data (30-m spatial resolution) were obtained and processed using the Google Earth Engine (GEE) platform (Dong et al., 2016; Kelley, Pitcher & Bacon, 2018). The GEE derived Landsat Annual TOA Reflectance Composite data was created by taking the median value from image bands from the target year, plus or minus one year (Midekisa et al., 2017). TOA corrected Landsat data have been extensively used for land cover mapping and classification in different tropical ecosystems, including tropical Asia (Ramdani, Moffiet & Hino, 2014; Johnson & Iizuka, 2016; Dong et al., 2015; Pimple et al., 2018).

#### ALOS L-band data

L-Band ALOS PALSAR data (25-m spatial resolution) were obtained in 2016 from Japan Aerospace Exploration Agency. These data have dual polarization HH (horizontal transmit, horizontal receive) and HV (horizontal transmit, vertical receive). These data are provided as digital numbers (DN). In the backscatter values (*σ*^0^) (Eq. (1)), the DN values of both HH and HV were converted to the normalized radar cross section (Avtar et al., 2013; Singh et al., 2017a):

(1) \begin{eqnarray*}& & \sigma 0=10\bullet log10(DN)^{2}+CF.\end{eqnarray*}

An enhanced Lee filter was implemented using a 3x3 kernel size to reduce the noise caused by speckles (Morel et al., 2011). These backscatter values are strongly associated with forest structural properties, including canopy cover (Mitchard et al., 2011; Deus, 2016; Singh et al., 2017a). The backscatter HH and HV values were then used to compute two radar-based indices- Radar Forest Degradation Index (RFDI) and the ratio of HH and HV (HH/HV) (Eq. (2)).

(2) \begin{eqnarray*}& & RFDI=(HH-HV)/(HH+HV),\end{eqnarray*}

This index varies from 0 to 1, with values closer to 1 indicating a more open canopy and thus higher levels of degradation (Singh et al., 2017a; Singh et al., 2018). This index was used to map different forest cover types in Gabon (Mitchard et al., 2012) and to distinguish forest patches that had undergone varying levels of degradation in Laos (Singh et al., 2017a). This index was also used to differentiate and map cashew plantations and forests in a protected area in Cambodia (Singh et al., 2018) and for land cover mapping in northern Tanzania (Deus, 2016). The HH/HV ratio has been used for land cover classification (Deus, 2016; Ali, Qazi & Aslam, 2018) and was proven useful in differentiating between forest and non-forested areas in the Riau province in Indonesia (Quegan et al., 2014).

#### LiDAR

The LiDAR data were collected over the study area in 2016 using a Leica ALS60 laser system and a 40 megapixel Leica RCD105 medium-format camera in an external pod mounted on the left skid of a Eurocopter AS350 B2 helicopter. These data have a density of 15 points/m^2^. To achieve requisite accuracy and point density, flights were at altitudes of 800–1,000 m above-ground level at a speed of 80 knots, with the ALS70 configured to Multipulse in Air (MPiA). The pulse rate was 500 kHz with a scan angle of 45° from nadir and a swath side-lap of 50% (i.e., almost all terrain was scanned twice from different angles). Aircraft attitude was measured by a Honeywell CUS6 IMU at a rate of 200 kHz and positional data was logged at 2 Hz using a survey-grade L1/L2 GNSS receiver mounted in the tail rotor assembly (Evans, 2016). The processing of these data to obtain a canopy height model (CHM) at 25m spatial resolution was carried out as done by Singh et al. (2018). CHMs provide a 3D representation of tree canopy heights. These were obtained by deriving a digital elevation model (DEM) from ground LiDAR returns and the vegetation/non-ground returns were used to a digital surface model (DSM). These were subtracted from each other to obtain a CHM (Singh et al., 2015). CHMs have been extensively used for tree species identification and biomass mapping and changes in Cambodia (Singh et al., 2015; Singh et al., 2016; Singh et al., 2018).

### Statistical data analysis

#### Spectral and structural properties evaluation for the different land use types

Prior to any statistical analysis, the Landsat data were resampled to 25-m to be at the same spatial resolution as ALOS PALSAR and LiDAR-derived data. All the Landsat bands were used in the analysis to enable better discrimination between the different land cover classes (Bhagwat et al., 2017). Reflectance values for the Landsat bands, ALOS-derived variables and LiDAR CHM were extracted for the geolocations of the different land use types collected in the field. Kruskal-Wallis (KW) tests were applied using the using the R statistical software package, version 3.3.2 (R Core Team, 2013) to examine whether the Landsat based spectral and ALOS and LiDAR-derived forest structure variables (HH, HV, HH/HV, RFDI and LiDAR CHM) varied across the different land cover classes, including cashew plantations. This is a nonparametric test that does not need the assumption of normally distributed data (Gaveau et al., 2009; Singh et al., 2017a). The “pgirmess” package of R (Giraudoux, 2018) was used to carry out post hoc analysis to identify which land use types were significantly different in terms of their spectral and structural properties as done by Rembold et al. (2017) and Singh et al. (2017a).

#### Land cover mapping

Three commonly used ML algorithms for tropical land cover classification, SVM, RF, and ANN were employed for classification purposes. The training used 70% of the geolocation data and the other 30% for testing the models (Rogan et al., 2008; Wang et al., 2015). Classification metrics including overall accuracy, kappa coefficients, user’s and producer’s accuracy were computed from the testing data. Overall accuracy computes how classified pixels compare with the field collected test data. Producer’s accuracy measures how well the real world classes can be classified. User’s accuracy quantifies the likelihood of a classified pixel matching the land cover type of its corresponding real-world location (Rwanga & Ndambuki, 2017). Additionally, sensitivity and specificity were calculated using Eq. (3) to evaluate the ability of the different ML and remote sensing data combinations to discriminate between specific land cover classes (Silva et al., 2017), in this case, cashew plantations from other land use types, based on their geometric mean (G).

(3) \begin{eqnarray*}& & G=\sqrt{}(sensitivity\ast specificity),\end{eqnarray*}

Classification errors can result in the area and extent of a class to be overestimated or underestimated (Alatorre et al., 2011). Sensitivity is the proportion of true positives classified by the algorithm and specificity is the proportion of true negatives. In many cases, high sensitivity is associated with low specificity and vice versa. Computing the geometric mean allows us to combine the two and balance between detecting and not detecting a certain land cover class (Silva et al., 2017; Silva, Bacao & Caetano, 2017).

#### Support vector machine

SVM is a supervised nonparametric statistical learning method. The SVM aims to find a hyperplane that optimally separates linearly-separable classes (Singh et al., 2015). In the simplest form, SVMs are binary classifiers that assign the given test sample to one of two possible classes. The SVM algorithm is extended to non-linearly separable classes by mapping samples in the feature space to a higher dimensional feature space using a kernel function. Data projection via hyperplanes allows the algorithm to discriminate between the different land use classes (Kanniah et al., 2015). SVMs are particularly appealing in remote sensing due to their ability to successfully handle small training datasets, often producing higher classification accuracy than traditional methods (Maryantica & Lin, 2017). It is also a non-parametric algorithm, i.e., it is not influenced by the underlying data distribution (Tiwari et al., 2016).

The SVM algorithm was implemented using the *caret* package of the R programming language (Kuhn, 2015). The SVM algorithm in the “caret package” is implemented via the kernlab package of the R programming language (Karatzoglou et al., 2004). The radial bias function (RBF) kernel which has been extensively used for land cover applications (Thanh Noi & Kappas, 2018) was used for this research. RBF-kernel based SVM has produced robust results with complex land cover related datasets (Ghosh & Joshi, 2014) and use of SAR data for tropical forest mapping in Indonesia (Trisasongko et al., 2017). The use of the radial bias kernel function requires tuning of two parameters: cost of constraints violation (C) and gamma. The former accounts for the over-fitting of the model while the latter controls the shape of the hyperplane. A cost function value (C) of 10 and a gamma value of 0.1 were finally used as tuning parameters (Wang et al., 2015; Chuang & Shiu, 2016). A 10-fold cross-validation repeated three times for the model building (Ghosh & Joshi, 2014). The SVM tuning parameters are tabulated in Table 2.

**Table 2:** Parameters of the classifiers used in building the classification models.

Classifier	Parameter		
ANN	size	decay	rang
	10	5e-6	0.1
RF	mtry	ntree	
	6	500	
SVM	kernel	cost	gamma
	“radial”	10	0.1
	“optimal”	9	1

#### Random forests

RF is a decision tree-based ML algorithm. Single decision tree models are built through recursive partitioning, wherein the response variable is iteratively divided into groups sequentially with group ’purity’ increasing with each division. RF models fit multiple decision trees to input data using a random subset of the input variables for each tree constructed. An average derived from the multitude of trees is used to form the predictive model (Singh et al., 2015). RF models are non-parametric, handle a large number of correlated input variables and skewed data and prevent overfitting. This makes RF models very useful for mapping heterogeneous landscapes (Attarchi & Gloaguen, 2014). For classification purposes, an ensemble of individual decision- tree classifiers are created and these are combined using a majority voting scheme (Singh et al., 2018). The RF classifier of this study was implemented using the “caret” package of the R programming language (Kuhn et al., 2016). Caret package implements the RF algorithm using the “randomForest” package (Liaw & Wiener, 2002). Since classification accuracy is sensitive to tuning parameters, the number of trees (n) was fixed at a default value of 500 (Li et al., 2016; Raczko & Zagajewski, 2017). The other tuning parameters were identified using the in-built *tuneGrid* function of the algorithm. The *tuneGrid* is a function that searches for optimal mtry values (the number of random predictors used for splitting at a node) given the data (Ghosh & Joshi, 2014). This parameter tuning process was performed following a 3 time repeated 10-fold cross-validation process (Chuang & Shiu, 2016; Ghosh, Sharma & Joshi, 2014). The RF tuning parameters are tabulated in Table 2.

#### Artificial neural networks

ANN is a biologically inspired ML algorithm that has been extensively used for building predictive regression models with nonlinear ecological data (Foody & Cutler, 2006). Multi-Layer Perceptrons (MLPs) are the most common type of ANN used for remote sensing studies and were used for this study. MLP networks use a back propagation learning principle and one hidden layer, and are useful for modeling non-linear relationships (Jensen, Fang & Minhe, 1999). This setup is typically composed of an input layer (for feeding in predictors), an output layer (a response variable) and one hidden layer which accounts for the non-linearities in the data (Liu et al., 2017b). The model is trained by predicting the response from patterns learned from a training data set (Chambers et al., 2016). By comparing the current output layer to the desired output response, the difference between the two can be obtained and used to adjust weights within the network. The goal is to find weights that produce results that closely resemble the target response. This iterative modelling process is repeated until the predicted response values meet a given level of accuracy (Ingram, Dawson & Whittaker, 2005). The MLP based ANN was implemented using the remote sensing data as predictors through the *nnet*” package of R (Venables & Ripley, 1997). The nnet algorithm simulates feed-forward neural networks with a single hidden layer with a backpropagation algorithm as the training algorithm (Werbos, 1994). Implementation of the algorithm needs the tuning of three important parameters, size, decay and maxit. The size parameter sets the number of units in the hidden layer and needs to be tuned. The decay parameter controls the weight decay, and maxit sets the maximum number of iterations; they are both left at their default values (Li et al., 2016; Raczko & Zagajewski, 2017). A logistic activate/transfer function and the quasi-Newton optimization algorithm that does not use the parameters, such as learning rate and momentum, were used (Venables & Ripley, 1997). The tuning parameters are tabulated in Table 2.

## Results

### Spectral and structural properties of the different land use types

**Figure 2:**
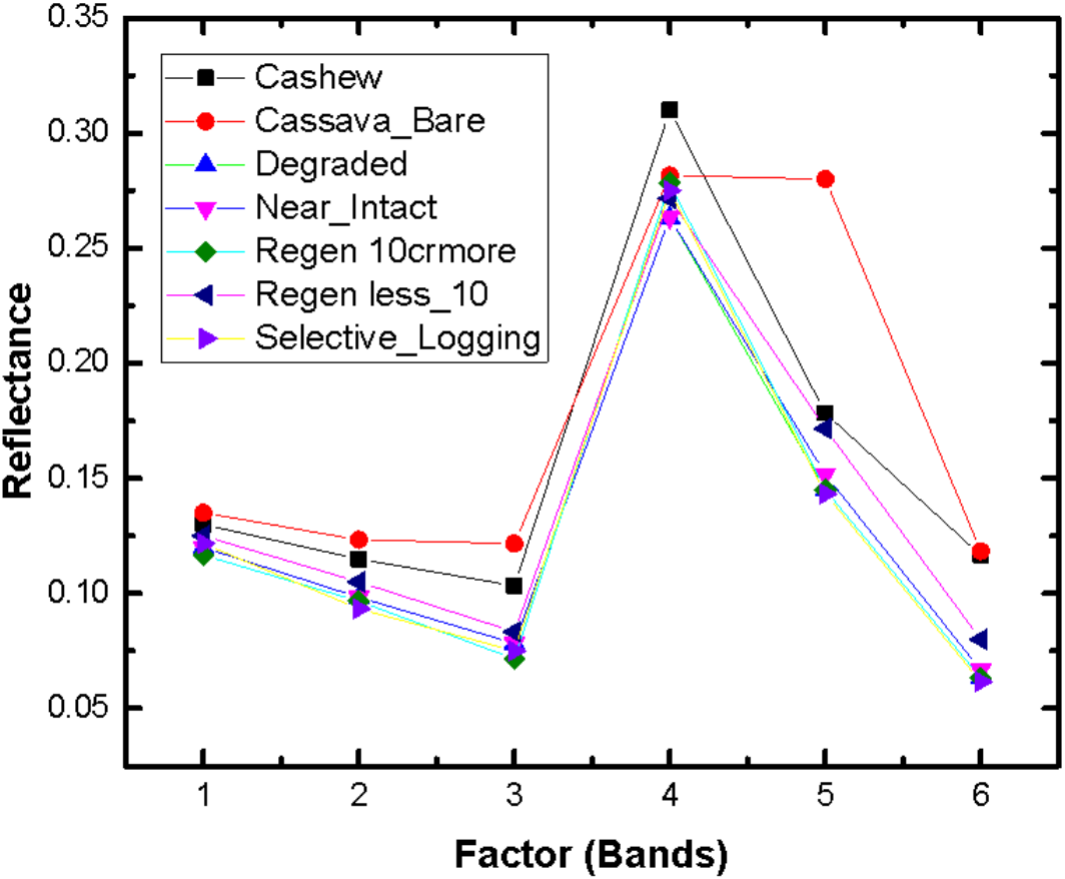
Spectral Behaviour of the Different Landcover Classes in PKNP.

The reflectance curves of different land use classes across different Landsat bands are shown in Fig. 2. It indicates that cashew plantations had the highest reflectance (out of all land cover types) in band 4. The reflectance curves of different natural forests were very close to each other, especially in band 3, 4 and 5.

KW tests revealed that in band 3, cashew plantations were significantly different from all the land cover classes, except cassava and selective logging (KW = 113.8, *p* < 0.05). The degraded forests were significantly different from the virtually intact forests only. The near-intact forests, regenerating forests and selectively logged forests were not significantly different from each other in band 3. In band 4 and 5, the cashew plantations were significantly different from all land cover classes, except the cassava plantations (KW = 68.3, *p* < 0.001). The natural forest types: degraded forests, regenerating forests (both more than and less than 10 years), near-intact and selectively logged forests were not significantly different from each other in band 4. In band 5, the degraded forests varied significantly from all natural forest types, except the selectively logged forests.

**Figure 3:**
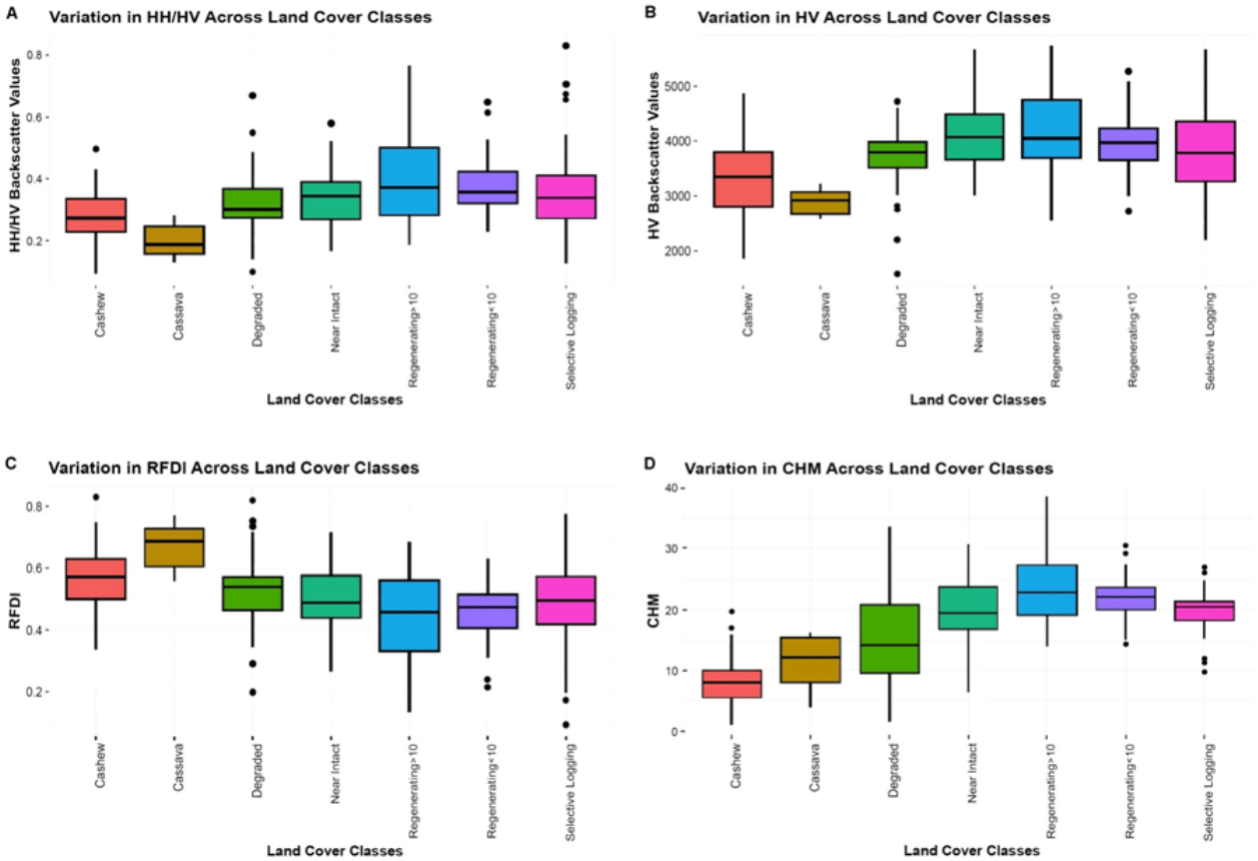
(A) Varaition in HH/HV across different land cover classes, (B) variation in HV across the different landcover classes, (C) variation in RFDI across different land cover classes, (D) variation in CHM across different land cover classes.

KW test also revealed that the HH backscatter values did not vary significantly across the different land use classes. However, as Fig. 3 shows, the other structural metrics- backscatter HV, HH/HV ratio, RFDI and LiDAR-derived CHM varied significantly across the different land use classes (KW = 40.58, 35.2, 35.7, 109.9, respectively, *p* < 0.005).

Post-hoc analysis revealed that the HV backscatter varied significantly between both cashew plantations and near intact forests and between cashew plantations and regenerating forests (which had been regenerating for both more and less than ten years). The HH/HV ratio and RFDI varied significantly between the cashew plantations and regenerating forests. These metrics varied significantly between cassava plantations, near intact forests, regenerating forests and selectively logged forests. Cashew plantations had a mean RFDI value of 0.57 while the regenerating forests had average values of 0.43 and 0.45 respectively for the forests that had been regenerating for more than 10 years and less than 10 years. LiDAR-derived heights (from the CHM) were significantly different for cashew plantations and the other land cover types in question. Out of all the land cover types, cashew plantations had the lowest average values (mean = 8.4, sd = 3.9) while the average vertical canopy heights of the other classes varied from 11 m–23 m.

### Comparison of classification results with different datasets and ML techniques

Of all the RS datasets combinations considered, the Landsat-only band data performed the worst with all ML algorithms. The overall accuracy of the kernel based SVM model was 56.0% when all the spectral bands were considered. With RF and ANN, the overall classification accuracy was 62.0% and 28.5% respectively (Table 3). The kappa coefficients of the SVM, RF and ANN models were 0.45, 0.54 and 0.16 respectively. However, the RF model also produced a geometric mean of 0.9 for the cashew plantations, indicating a strong ability to discriminate cashew plantations. In addition to the Landsat spectral bands, the four ALOS derived products were included and the three ML techniques were implemented on these. The overall accuracies obtained with SVM, RF and ANN were 95.0%, 63.0% and 33.0% respectively. The kappa coefficients of the SVM, RF and ANN models were 0.94, 0.54 and 0.21 respectively. The SVM model had a G-value of 1 for cashew plantations while the RF model produced a G-value of 0.82. The SVM variable importance analysis revealed that out of all the ALOS-PALSAR variables included in the mapping process, the HV band and RFDI were most useful in discriminating between the different land use classes.

**Table 3:** Overall accuracy of the different ML models and their corresponding Kappa values.

**Different RS datasets used**	**SVM**	**RF**	**ANN**
Landsat spectral bands	56.0% (0.45)	62.0% (0.54)	28.5% (0.16)
Landsat spectral bands + ALOS metrics	95.0% (0.94)	63.0% (0.54)	33.0% (0.21)
Landsat spectral bands + ALOS metrics LiDAR CHM	98.0% (0.98)	66.3% (0.59)	31.0% (0.19)

The highest overall accuracy was obtained when LiDAR CHM data were included along with the Landsat and ALOS data bands. The overall accuracies obtained with SVM, RF and ANN were 98.0%, 66.3% and 31.0% respectively. The kappa coefficients of the SVM, RF and ANN models were 0.98, 0.59 and 0.19 respectively. The SVM model had a G-value of 1 for cashew plantations while the RF model produced a G-value of 0.72. The SVM variable importance analysis revealed that LiDAR CHM was more important than the ALOS-PALSAR and Landsat variables included in the mapping process.

### Land cover map

**Figure 4:**
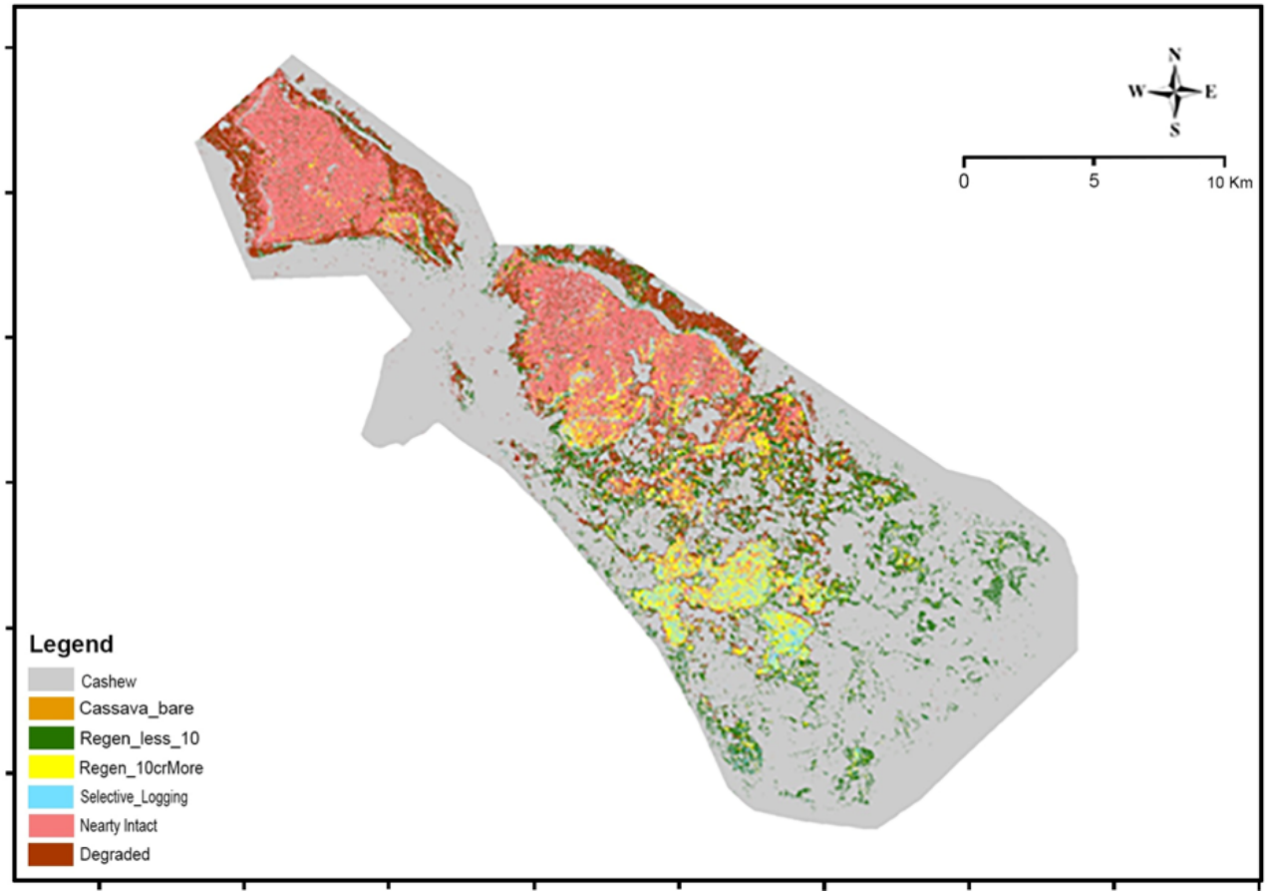
Land Cover Map of PKNP.

A combination of Landsat-ALOS data within a framework of an SVM classification algorithm had an overall accuracy of 95.0%. However, the inclusion of LiDAR-derived canopy heights improved the overall classification accuracy to 98.0%. This model was thus used for producing a land cover map of PKNP (Fig. 4). The producer and user accuracies of the SVM model which utilized the Landsat, ALOS and LiDAR data have been tabulated in Table 4. All the land use classes were identified accurately. The analysis also revealed that more than 60.0% of PKNP is dominated by cashew plantations. Nearly intact forests which cover 13.3% are clustered in the northern part of PKNP, which is relatively inaccessible.

**Table 4:** Comparison of producer’s and user’s accuracy levels for Landsat-ALOS-LiDAR data SVM model.

	**Cashew**	**Bare Earth**	**Regenerating for less than 10 years**	**Regenerating for more than 10 years**	**Selective logging**	**Lightly logged forests**	**Degraded Forests**
% User’s accuracies	100	100	100	100	100	100	87.5
% Producer accuracies	100	100	100	94	100	100	100

## Discussion

Landsat spectral data models have been used for both mapping land cover and for identifying land cover change across different tropical forest ecosystems (Connette et al., 2016; Hansen et al., 2016; Bhagwat et al., 2017; Hurni et al., 2017; Shen, Li & Wei, 2017). The ability of these data to distinguish between different land cover/forest types varies considerably across different tropical ecosystems. In this study revealed that the separability between the different land cover classes (especially between the different natural forests and cashew plantations) is limited across the Landsat bands. The Landsat spectral band model utilized for land cover mapping had an overall accuracy of 68.0%. This is similar to the overall accuracy of a Landsat spectral data only map developed using an ML model developed to map 32 different land use classes in Costa Rica (Sesnie et al., 2008). The relatively low overall classification accuracy of the Landsat model, when used in isolation, can be attributed to low spectral separability of different tropical forest/land cover types in Landsat imagery (Tottrup, 2004). This research is the first of its kind which has identified low spectral separability exists between natural forest types, and between cashew plantations and natural forests using Landsat-only models. This is cognizant with the existing body of literature. The phenomenon of lower spectral separability between plantation monocultures and natural forests has been observed in other plantation types such as rubber (Li & Fox, 2012), oil palm plantations (Broich et al., 2011) and teak (Win, Reiji & Shinya, 2009).

To mitigate this weakness, in several applications, ALOS L-band data and Landsat spectral bands have been used jointly to map different tropical forest types (De Alban et al., 2018) including plantation monocultures (Kou et al., 2015; Chen et al., 2018). HH and HV backscatter values are known to be influenced by the structure of monocultures such as oil palm and rubber plantations (Torbick et al., 2017). This research too discovered that inclusion of backscatter derived metrics helps improve the classification accuracy and distinguish between the forests that had undergone varying levels of degradation and cashew plantations. The inclusion of ALOS derived metrics produced higher classification accuracy for mapping the successional forests of the Brazilian Amazon than Landsat-only model (Li et al., 2011). A combination of Landsat spectral bands and ALOS L-band derived metrics (including RFDI) were used within an SVM model to differentiate between different land use classes in northern Tanzania with a high level of accuracy (Deus, 2016). A combination of Landsat spectral bands and ALOS data previously outperformed individual sensor data models for mapping different land use types (including human-modified landscapes such as plantations) in southern Myanmar (De Alban et al., 2018). The research also establishes that the HH-band of the ALOS data did not vary appreciably between the different land cover classes. Previous research discovered that HV bands can distinguish between different tropical land cover types better than the HH backscatter values (Dong et al., 2014; Singh et al., 2017b). The HV backscatter was able to discriminate cashew plantations from intact and regenerating forests. While this is the first time these metrics have been used for mapping cashew plantations, previous research has established that HV backscatter values are sensitive to and can help differentiate oil palm plantations from natural forests in Cameroon (Li et al., 2015). HV backscatter was found to be sensitive to both oil palm and rubber plantations in Kalimantan and Myanmar respectively (Torbick et al., 2017). This, in turn, may be attributed to the fact that HV backscatter interacts closely with the vertical structure of the trees (Mitchard et al., 2011) which makes it sensitive to the vertical structural differences between plantation monocultures and natural forests. Derived products, notably the HH/HV ratio, have distinguished woody plantations such as rubber from other forests in SE Asia (Win, Reiji & Shinya, 2009; Dong et al., 2014). In this study too, the HH/HV ratio of the cashew and cassava plantations was different from regenerating, selectively logged and intact natural forests. It was also discovered that in addition to the HV backscatter values, RFDI were important variables for mapping land cover types. RFDI was included as it can help differentiate between different forest/vegetation types in the tropics (Mitchard et al., 2012). This is the first time RFDI was used to map a plantation monoculture. This research establishes that RFDI values vary significantly between cashew plantations and natural forests. Previously, RFDI was used successfully to differentiate between forests that had undergone varying levels of degradation in Laos (Singh et al., 2017a), to distinguish forest types in northern Tanzania (Deus, 2016) and forests and non-forests (Ningthoujam et al., 2016).

In this research, the inclusion of structural attributes (derived from LiDAR and ALOS L-Band data) improved the classification accuracy of the land cover models considerably (when used in conjunction with the SVM algorithm). Out of all the data, the LiDAR-derived canopy height was most sensitive to the differences between the land cover classes. LiDAR derived heights too were found to be significantly different between cashew plantations and natural forests, indicating that the former are structurally different from natural forests. LiDAR derived heights were previously used to differentiate between natural forests and cashew plantations accurately in the PKNP (Singh et al., 2018) and forests that have undergone varying levels of degradation in southern Laos (Singh et al., 2017a). LiDAR-derived vertical height estimates also proved effective in differentiating between different types of tropical moist forests in the Neotropics (Kennel et al., 2013).

The model which contained both ALOS and LiDAR-derived metrics had the highest overall accuracy of 98.0% although the Landsat-ALOS model had an overall accuracy of 95.0% with SVM classification. The RF model displayed a robust ability to identify cashew plantations even with the Landsat-only data (although the overall accuracy was much lower in this case). Both SVM and RF are known to outperform other classifiers for mapping and differentiating between different tropical forest types (Kennel et al., 2013). SVM models, especially give better results than other classifiers (including ANN) when used with smaller training sets and multiple RS band data (Pal & Mather, 2005). Three supervised classification methods, Maximum Likelihood Classification (MLC), SVM and ANN were tested to carry out classification of an urban ecosystem in Mexico. In this study, SVMs classification method performed better than both ANN and MLC. Land cover mapping of a complex landscape was conducted using three machine learning algorithms (RF, SVM, and ANN). SVM produced average overall accuracy of 86.63% while ANN models produced average overall accuracy of 73.55% (Li et al., 2016). Both SVM and RF models gave better classification results with multiple RS data sources as compared to other classifiers, including ANN (Xie et al., 2019). Owing to their ability to outperform more traditional classifiers for land use mapping in the tropics, SVM models have been used for differentiating between forest and land cover types in a number of tropical Asian ecosystems (Kanniah et al., 2015; Tiwari et al., 2016). SVM based classifiers were used to map healthy mangroves and differentiate them from other land use types in the Bay of Bengal region (Singh et al., 2014) and for land cover mapping in Vietnam (Thanh Noi & Kappas, 2018).

## Conclusions

The problem of cashew plantation encroachment in PKNP is a microcosm of the challenges faced by the protected areas of SE Asia. This study highlights the potential to harness multi-sensor data and different ML algorithms to distinguish between and map forests and plantations across a heterogeneous landscape. While LiDAR data can produce highly accurate classifications, owing to the expense, these data may be difficult to obtain for conservation managers in tropical Asia. This research used freely available Landsat and ALOS data and helped identify how these could be used to give an overall accuracy results comparable to the model that included the commercially available LiDAR data. Future research will undertake similar mapping techniques in other protected areas of tropical Asia to quantify the magnitude of encroachment by different plantations.

##  Supplemental Information

10.7717/peerj.7841/supp-1 Supplemental Information 1 Spectral and Structural Behavious of Land Cover Classes

## Additional Information and Declarations

### Data Availability

The following information was supplied regarding data availability: The raw data is available at Github: https://github.com/Jojo666/PKNP-Data.
